# A comparison of concurrent chemoradiotherapy and radiotherapy in Chinese patients with locally advanced cervical carcinoma: a multi-center study

**DOI:** 10.1186/1748-717X-9-212

**Published:** 2014-09-22

**Authors:** Zhijie Li, Shuyan Yang, Lei Liu, Shiyu Han

**Affiliations:** Department of Gynaecology and Obstetrics, Fourth Affiliated Hospital of Harbin Medical University, No.37 Yiyuan Street, Nangang District, Harbin, Heilongjiang Province 150001 China

**Keywords:** Advanced cervical carcinoma, Concurrent chemoradiation, Radiotherapy

## Abstract

**Background:**

We investigated the efficacy of concurrent chemoradiotherapy (CCRT) over radiotherapy (RT) in Chinese patients with locally advanced cervical carcinoma.

**Patients and methods:**

Between January 2005 and January 2008, 192 patients with squamous cell carcinoma of the cervix were included in the study: 96 in arm A (CCRT with 20 mg/m^2^ cisplatin for 5 days) and 96 in arm B (RT). The overall response rate was the primary endpoint. The secondary endpoints included overall survival, progression-free survival, and toxicity.

**Results:**

The 5-year overall response rate was 67% and 53% for the CCRT and RT arms, respectively, and the difference was statistically significant, while the median overall survival was 68 months (range 3-85 months) and 61 months (range 4-83 months), respectively (*P* = 0.009). In addition, the median progression-free survival for CCRT was 62 months (range 3-83 months), whereas it was 51 months (range 4-81 months) for the RT arm (*P* = 0.025). The toxicity profile, both acute and late, was comparable in both arms.

**Conclusion:**

In summary, we demonstrate that CCRT was effective and better tolerated than RT alone in Chinese patients with locally advanced cervical carcinoma.

**Trial registration:**

Chinese Clinical Trials Register: ChiCTR-TRC-13003979.

## Introduction

Cervical carcinoma is an important cause of cancer-related death in women in developing countries, and thus, continues to pose a major threat to their health [1, 2]. It is the commonest malignancy among women in China and is widely prevalent all over the country. The 5-year overall survival rate mainly depends on the stage of the tumor [3, 4]. It varies from 98% in early stage disease to 10% in the most advanced stages [5]. In the last decade, survival has improved due to the development of new treatment strategies such as the combination of chemotherapy and radiation [6, 7].

Currently, a number of treatment options for patients with cervical carcinoma are available, including surgery [8, 9]; radiotherapy [10, 11]; chemotherapy such as carboplatin [12], cisplatin [13], paclitaxel [14], topotecan [15], gemcitabine [16], docetaxel [17], ifosfamide [18], 5-fluorouracil (5-FU) [19], irinotecan [20], mitomycin [21], and bevacizumab [22]; and hyperthermia [23], either as a single or combined modality [24, 25], wherein the choice of treatment aims at achieving the best results with the least morbidity.

Until 1999, the primary treatment for locally advanced cervical carcinoma was radiotherapy, where the tolerance of normal tissues limits the dose intensity [5, 26]. Recently, the results of a number of clinical trials have shown that concurrent chemoradiotherapy (CCRT) provides higher cure rates than radiotherapy alone [27–29]. Therefore, CCRT is now considered standard treatment for patients with tumors in stage IIB or higher [30, 31]. However, there is inadequate data about the efficacy and safety of CCRT in Chinese patients with locally advanced cervical carcinoma. Therefore, the primary aim of this randomized study was to prove the superiority of CCRT over radiotherapy (RT) alone in terms of survival, failure rate, and toxicity.

## Patients and methods

Between January 2005 and January 2008, 192 patients with a pathological diagnosis of stage IIB-IIIB squamous cell carcinoma of the cervix as per the International Federation of Gynecology and Obstetrics (FIGO) criteria, were recruited to participate in this study. To be eligible, patients had to be between the ages of 20 and 65 years, with a Karnofsky performance status ≥70, hemoglobin ≥10 g/dL, leukocyte count ≥3000/mm^3^, absolute neutrophil count ≥1500/mm^3^, platelets ≥100,000/mm^3^, creatinine clearance ≥50 mL/min, and normal liver function test results. In addition, informed consent documents were signed by the patients. Patients with nonsquamous histology, para-aortic lymph nodes, distant metastases, or synchronous/metachronous malignancy were excluded. The study was approved by the institutional ethics committees of Third Affiliated Hospital and the Fourth Affiliated Hospital of Harbin Medical University with permission number (KY2005-01 and RR2004-12).

The randomization code was generated using a computerized number generator through the stratified block randomization method of the SAS package (SAS Institute Inc., Cary, North Carolina, USA) by a statistician with no clinical involvement in this trial. After qualifying, patients were assigned to 2 treatment arms: CCRT (arm A) or RT (arm B) by investigators at each center. The allocation was concealed in sequentially numbered, opaque, sealed envelopes containing the randomization assignments. In addition, all the outcome assessors and data analysts were blinded in this study.

The plan was to conduct the research in the Third Affiliated Hospital and the Fourth Affiliated Hospital of Harbin Medical University. In preparing for this research, we identified that these 2 centers had offered CCRT or RT treatment to 192 patients between January 2005 and January 2008. The individuals who were accepted for CCRT and RT were informed about the research and given an information sheet and signed informed consent. After the clinical assessment, patients were randomized to receive CCRT or RT, which was delivered by fully qualified therapists.

The treatment schedule consisted of external-beam RT to the pelvic region delivered with 15-MV X-rays. Pelvic radiation was given in daily fractions of 2 Gy for 5 days/week with a four-field technique (anterior and posterior portals: 0.6 Gy, lateral portals: 0.4 Gy), up to a total dose of 46 Gy/23 fractions. The upper border of the pelvic portal was at the L4-L5 junction. The lower border was at the lowest part of the obturator foramen, which was modified according to the vaginal extent of the disease. The lateral borders were kept 1.5-2 cm lateral to the bony pelvis. When the four-field box technique was used, the anterior or posterior borders of the lateral portals were kept at the cortex of the pubic symphysis and in the middle of the S2 vertebrae, respectively, and verified with a computed tomography (CT) scan with markers and extended posteriorly, if needed.

A cervical boost was given using the X-ray arch technique or high-dose-rate (HDR) intracavitary brachytherapy at a total dose of 10 Gy. Brachytherapy was conducted using an iridium-192 source and consisted of 2 separate endocavitary insertions, with an interval of at least 48 h, with the help of Fletcher-Suit after-loading applicators (Radium Chemical Company, New York, USA) and vaginal cylinders. As per the recommendations of the International Commission on Radiation Units and Measurements (ICRU 38), orthogonal films (at 45° and 315°) were taken to verify the placement of the applicators, to evaluate doses, and to deliver the dosimetric plan. Acceptable doses for the bladder and rectum were equivalent to 75% and 70%, respectively, of the dose received at point A on the radiographs; manual and graphic optimization was allowed.

In addition to RT, patients in the CCRT arm also received 20 mg/m^2^ cisplatin for 5 days, at 21-day intervals for a total of five cycles.

All measurable lesions were evaluated for tumor response according to the Response Evaluation Criteria in Solid Tumors (RECIST 1.0) [32]. All radiological assessments were confirmed by extratumoral reviews. Toxicity was graded according to the National Cancer Institute Common Toxicity Criteria for Adverse Events (AEs) (CTCAE) version 3.0 [33].

An intention to treat analysis was performed. Sample size was calculated with an expected 15% difference between the 2 arms. Overall survival was calculated from the day of randomization to the day of death. Data on patients who were alive were censored on the date on which they were last known to be alive. Relapse-free survival was computed from the date of randomization to the date of relapse, death or completion of follow-up, whichever occurred first. Data on patients who were alive and relapse-free were censored at the time of the last follow-up visit. Overall survival and relapse-free survival rates were calculated by the Kaplan–Meier method and a *P* ≤ 0.05 was considered statistically significant. All *P* values were obtained from 2-tailed t-tests.

## Results

**Figure 1:**
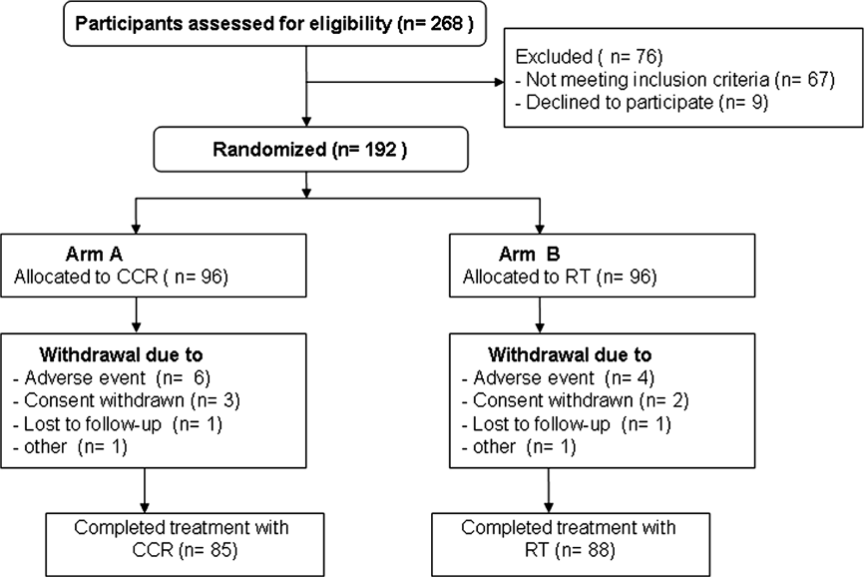
**Flow of participants through trial.**

In this study, 268 participants were initially screened. Of these 268 patients, 76 subjects were excluded. Of these 76 patients, 67 did not meet study criteria, and 9 declined to participate. The remaining 192 patients (CCRT, n = 96; RT, n = 96) were entered into the study. 173 participants completed the efficacy assessment. 19 patients withdrew from the study. The major reasons for withdrawal were AEs, withdrawn consent, and failure to follow-up (Figure 1). The baseline characteristics of the patients were similar in the 2 treatment arms (Table 1).

**Table 1:** **Baseline characteristics of participants at trial entry: ITT population**

	Variable	CCR (n = 96)	RT (n = 96)	***P***value
Age, yrs: mean (SD)		46.5 (14.1)	46.2 (13.9)	0.9
Race	Asian (Chinese)	96 (100.0%)	96 (100.0%)	1.0
FIGO stage				
	IIB	39 (40.6%)	41 (42.7%)	0.8
	IIIA	32 (33.3%)	31 (32.3%)	0.9
	IIIB	25 (26.0%)	24 (25.0%)	0.9
Tumor size (cm)				
mean (SD)				
	Median (range)	4.1 (2.3-6.4)	4.0 (2.2-6.3)	0.9
	Average	4.5 (1.9)	4.4 (1.8)	0.7
Deaths due to cervical carcinoma (n)		24 (25.0%)	33 (34.4%)	0.2

**Note:** ITT, intent-to-treat; CCR, concurrent chemoradiation; RT, over radiotherapy; yrs, years; SD, standard deviation; FIGO, International Federation of Gynecology and Obstetrics.

The median number of treatment cycles was three (range 1-5) for CCRT, the duration of which was 11 weeks; and three (range 1-5) for RT, the duration of which was 10 weeks. The main reasons for discontinuation of treatment were AEs [CCRT versus RT, 6/96 (6.2%) versus 4/96 (4.2%)], consent withdrawal [3/96 (3.1%) versus 2/96 (2.1%)], failure to follow-up [1/96 (1.0%) versus 1/96 (1.0%)], and other reasons [1/96 (1.0%) versus 1/96 (1.0%)].

**Figure 2:**
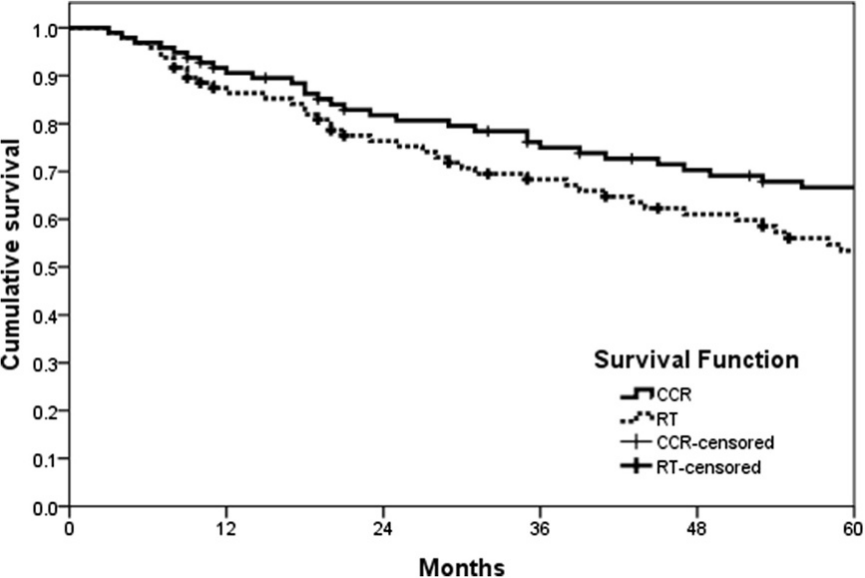
**Overall survival.**

**Figure 3:**
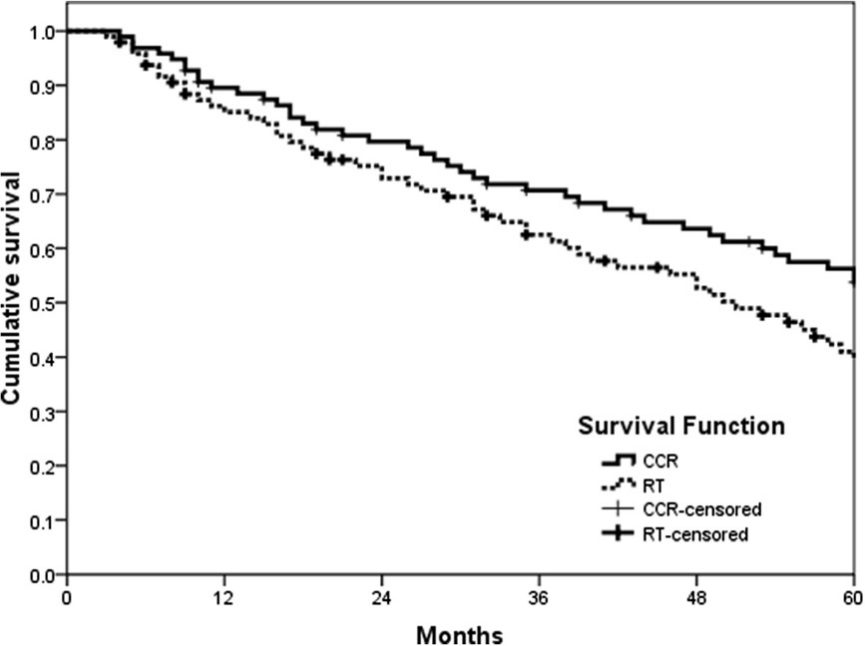
**Progression-free survival.**

The overall response rate as determined by the RECIST criteria, was 67% for CCRT (n = 96) and 53% for RT (n = 96). The difference was statistically significant (*P* < 0.05; Table 2). The median overall survival was 68 months (range 3-85 months) and 61 months (range 4-83 months) for the CCRT and RT arms (*P* = 0.009), respectively (Figure 2). In addition, the median progression-free survival was 62 months and 51 months for the CCRT (range 3-83 months) and RT arms (range 4-81 months), respectively (*P* = 0.025; Figure 3).

**Table 2:** **Summary of adverse events**

Adverse events	CCR (n = 96)	RT (n = 96)
	G3/4 (≥G3) (%)	G3/4 (≥G3) (%)
Leukopenia	8/3 (11%)	4/3 (7%)
Thrombocytopenia	2/0 (2%)	0/0 (0%)
Nausea	4/1 (5%)	2/0 (2%)
Diarrhea	11/0 (11%)	4/0 (4%)
Anaemia	3/0 (3%)	1/0 (1%)

AEs that occurred in each group are shown in Table 2. 96 CCRT and 96 RT patients were included. The incidence of major hematological toxicities was higher with CCRT than with RT. Grade 3 or 4 leukopenia was observed in 11/96 (11%) of patients treated with CCRT versus 7/96 (7%) of patients treated with RT, while the corresponding incidences of thrombocytopenia were 2/96 (2%) versus 0/96 (0%), respectively. The most common grade 3 or 4 non-hematological toxicities were diarrhea (CCRT versus RT, 11/96(11%) versus 4/96 (4%)), nausea ((5/96) 5% versus 2/96 (2%)), and anorexia (3/96 (3%) versus 1/96 (1%)). There were no treatment-related deaths in either arm.

## Discussion

Radiotherapy has been used as the only therapeutic option for patients with locally advanced cervical cancer in the past [34]. As 20-50% of patients with stage IIB and 50-75% with stage III tumors suffered a relapse, additional treatments [32], including chemotherapy [12–22] and hyperthermia [23] were incorporated in order to enhance the effects of radiotherapy. For example, cisplatin-based chemotherapy with concurrent radiotherapy has now taken center stage in the therapy of locally advanced uterine carcinoma [35].

A previous study from the Gynecological Oncology Group compared patients who underwent radiotherapy with cisplatin treatment to those receiving hydroxyurea. The study included participants with stage IIB-IVA cervical carcinoma. The group treated with both radiotherapy and cisplatin had a higher rate (60%) of 5-year survival in contrast to the group with hydroxyurea (34%). The difference in survival was maintained after 10 years (53 and 34%, respectively; *P* < 0.01). In addition, patients with highly unfavorable pretreatment prognostic factors were closely monitored, especially those undergoing the 3-weekly regimen. The 3.5-year survival rate was comparable to previous results.

In this study, CCRT also had a favorable safety profile. The overall frequency of AEs was similar in both arms and most side effects were not severe. The frequency of both drug-related AEs and AEs of severe intensity was higher in the CCRT arm than in the RT arm.

A previous publication concluded that when bulky disease was defined as tumors larger than 4 cm, tumor size was also of prognostic importance in FIGO stage II cervical carcinomas [36]. In this study, there was no significant difference in the median and average sizes of tumor diameter between the 2 groups (*P* = 0.9 and *P* = 0.7 respectively), although tumor stages varied from IIB to IIIB. Tumor size was determined by pathological evaluation in the CCRT group and pretreatment MRI in the RT group.

Our study has several strengths. Firstly, the trial was randomized thereby reducing selection bias. Secondly, although there was no consensus regarding the dose appropriate for CCRT in Chinese patients with locally advanced cervical carcinoma, this trial suggested that our treatment was in the therapeutic range. Further studies with larger sample size and longer duration of CCRT are needed to further confirm the results of this study.

In conclusion, the results of this study show promising efficacy and a very acceptable toxicity profile for CCRT in Chinese patients with locally advanced cervical carcinoma. The follow-up period is still short, but the encouraging clinical and pathological results warrant further investigation.
